# Patient-Derived Glioma Models: From Patients to Dish to Animals

**DOI:** 10.3390/cells8101177

**Published:** 2019-09-30

**Authors:** Cintia Carla da Hora, Markus W. Schweiger, Thomas Wurdinger, Bakhos A. Tannous

**Affiliations:** 1 Experimental Therapeutics and Molecular Imaging Laboratory, Department of Neurology, Neuro-Oncology Division, Massachusetts General Hospital, Boston, MA 02129, USA; 2 Neuroscience Program, Harvard Medical School, Boston MA 02129, USA; 3 Department of Neurosurgery, Cancer Center Amsterdam, Brain Tumor Center Amsterdam, Amsterdam UMC, Vrije Universiteit Medical Center, 1081 HV Amsterdam, The Netherlands

**Keywords:** glioblastoma, cancer stem cells, patient-derived xenograft model, 3D cultures, organoids

## Abstract

Glioblastoma (GBM) is the most common and malignant primary brain tumor in adults associated with a poor survival. Current standard of care consists of surgical resection followed by radiation and chemotherapy. GBMs are highly heterogeneous, having a complex interaction among different cells within the tumor as well as the tumor microenvironment. One of the main challenges in the neuro-oncology field in general, and GBM in particular, is to find an optimum culture condition that maintains the molecular genotype and phenotype as well as heterogeneity of the original tumor in vitro and in vivo. Established cell lines were shown to be a poor model of the disease, failing to recapitulate the phenotype and harboring non-parental genotypic mutations. Given the growing understanding of GBM biology, the discovery of glioma cancer stem-like cells (GSCs), and their role in tumor formation and therapeutic resistance, scientists are turning more towards patient-derived cells and xenografts as a more representative model. In this review, we will discuss the current state of patient-derived GSCs and their xenografts; and provide an overview of different established models to study GBM biology and to identify novel therapeutics in the pre-clinical phase.

## 1. Introduction

Gliomas account for around 27% of primary and 80% of all malignant CNS tumors [1]. Glioblastoma (GBM), which comprises 54% of all gliomas, is the most malignant form. The Central Brain Tumor Registry (www.cbtrus.org) states that the five-year survival rate of adult patients with GBM is around 4.3%. Standard-of-care treatment involves maximal surgical resection of the tumor in combination with radiation and temozolomide chemotherapy; however, as the poor survival rate indicates, these treatments have not been effective in preventing disease progression [2,3,4,5]. GBMs are highly heterogeneous with complex interactions among different cells within as well as cells surrounding the tumor [6,7,8]. It is known that the majority of glioma cells do not have the capacity to recapitulate a phenocopy of the original tumor and that only a small subpopulation of neural stem-like cells within the tumor, called glioma stem cells (GSCs; or tumor initiating cells) have this ability upon xenotransplantation in immunocompromised nude mice [9,10]. These GSCs are notorious for their resistance to conventional therapy and are the source not only for tumor initiation, but also for recurrence [11]. Following current therapy for high-grade gliomas, most patients die within a year from a new secondary tumor foci forming within one centimeter of the resected area, enriched for GSCs [12]. These cells offer a clinically-relevant model to study GBM pathology and to find novel therapeutics, owing to their ability to self-renew, differentiate into multiple lineages, and recapitulate a phenocopy of the original tumor upon xenotransplantation [13,14,15]. Molecular classification of GBM identified three subtypes, classical, proneural (PN), and mesenchymal (MES) [6,16], with the latter displaying a more aggressive phenotype and pronounced radio/chemoresistance [17,18]. Single-cell RNA sequencing revealed that multiple subtypes can reside within the same tumor [8], confirming cellular heterogeneity in GBM. Further, PN tumors tend to shift toward a MES phenotype upon recurrence and in response to radiation/chemotherapies [17,18,19,20].

One of the main challenges in studying GBM is to develop an optimal culture condition that maintains the molecular heterogeneity of the primary tumor. Typically, once cells are dissociated from the patient tumor tissue, they are cultured as neurospheres in neurobasal medium supplemented with epidermal growth factor (EGF) and fibroblast growth factor (FGF2) [21]. These culture conditions maintain the phenotype and genotype of the primary tumor, and recapitulate the invasive property of GBM upon xenotransplantation in the mouse brain [22]. Patient-derived GSCs undergo differentiation by attaching to the culture dish once exposed to serum, thus losing the ability to form tumors in vivo [10,21,23,24]. In a recently published work, we showed that under acidic/hypoxic conditions, a small subset of these GSCs ‘escape’ this differentiation process and grow as floating cells. Studies on these subpopulations of cells revealed enhanced GSC properties with molecular and phenotypic features of the MES subtype, including resistance to radiation and targeted therapies, as well as a more aggressive tumor formation [25]. This model endorses the inherent intratumoral molecular heterogeneity in GBM, providing a valuable tool to study phenotypic plasticity. In this review, we discuss the current state of patient-derived GSCs and provide an overview of established models to investigate GBM progression and to facilitate pre-clinical identification of new therapeutics in vitro and in vivo.

## 2. In Vitro GBM Model

GBM grows in a three-dimensional (3D) configuration surrounded by and embedded in the tumor microenvironment. The exact composition of this highly heterogeneous environment can vary, but usually consists of the extracellular matrix, chemokines, growth promoting/inhibiting factors, and other cell types found in stromal tissue [26,27]. In this environment, GBM cells encounter hypoxic conditions and react to cues from surrounding tumor and non-tumor cells [28]. There is a strong clinical need to establish models mirroring the natural tumor environment. Traditional two-dimensional (2D) monolayer cell cultures fail to reflect these GBM environmental conditions. 2D-cultured cells differ considerably from cells growing in vivo with regards to cell-to-cell/matrix interaction, proliferation, and morphology, as well as signal transduction (Table 1) [29]. To achieve a more natural and holistic representation of the in vivo GBM microenvironment, 3D cultures have become the state-of-the-art approach in neuro-oncology.

**Figure 1:**
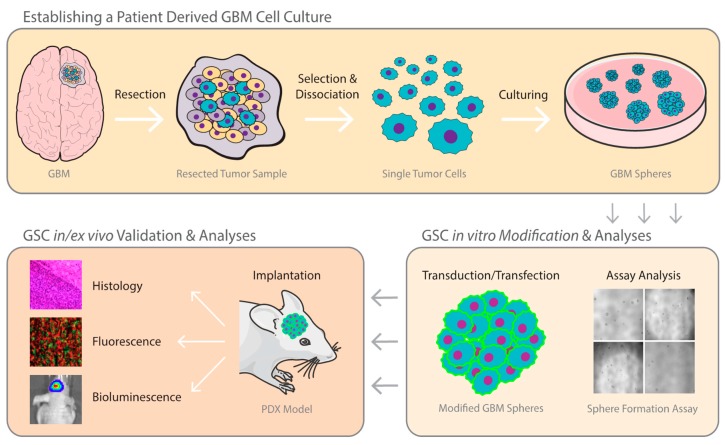
Glioblastoma model from patient to dish to animal. Typical workflow of establishing patient-derived glioblastoma (GBM) models. (Top) Tumor sample obtained from resective surgery is dissociated and single tumor cells are selected. Culturing with adequate growth conditions will facilitate the formation of GBM neurospheres. (Bottom Right) Obtained neurospheres can be tested for various properties, such as stemness, clonogenic potential, and viability; or can be engineered to express different proteins of interest or reporters for cell tracking. (Bottom Left) GBM neurospheres can be used to establish patient-derived xenografts (PDX) to study tumor biology and to test novel therapeutics by non-invasive monitoring of tumor volume with in vivo bioluminescence imaging or ex vivo fluorescence and histological analysis with Haematoxylin and Eosin staining. GSC: glioma stem cell.

A plethora of in vitro 3D culture systems, such as tumor derived organoids, organotypic multicellular spheroids, multicellular tumor spheroids or tumor-derived spheroids, have been established to better recapitulate the natural GBM heterogeneity and environment [30]. Each model system has been thoroughly described and comes with its own benefits and limitations with respect to validity, usability, reliability, and generalizability. Cancer-derived spheroids generally maintain the cellular heterogeneity, self-renewal capacity, and histological characteristics of the primary tumor. Importantly, these models mirror the multipotency of GSCs. Multipotency tests performed over time by multicolor phenotyping of purified subpopulations revealed that GSCs were responsible for creating phenotypic heterogeneity in vitro [31,32]. With this in mind, the following section will focus on the establishment, characterization, and utilization of tumor-derived spheroids, also known as neurospheres, summarized in Figure 1.

### 2.1. Establishing A GBM Stem Cell Line from Patient Tumor Tissue

A multitude of protocols for in vitro expansion of GSCs have been established [30], most of which exploit the exclusive properties of stem cells, not to simply survive, but to also proliferate in serum free media, often forming spheroid cell clusters. The source of cancer cells can vary, but GBM specimens are generally obtained from patients undergoing resective surgery. After approval of the Institutional Review Board, arrangements are made with the neurosurgery team to immediately place the resected tissue in cooled neural stem cell (NSC) medium supplemented with 10–15% antibiotics (streptomycin/penicillin). Resected specimens are transported on ice until cell isolation under sterile condition is initiated [33]. The tissue is first washed to remove excess debris and blood, and the tumor is cut and mechanically minced before digested with trypsin and deoxyribonuclease. After several washing and centrifugation steps, the digested tissue is titrated and passed through a cell strainer. Finally, cells are pelleted by centrifugation, resuspended, and cultured in Neurobasal medium supplemented with l-glutamine, B-27 supplement, human EGF, human FGF2, and penicillin [34]. It has been suggested that only cells with stem-like properties will form spheres and grow under these conditions [21,34]. Whether further purification of these cultures using specific surface markers is needed to obtain a pure population of GSCs remains to be a matter of a debate. CD133 (prominin-1) has long been regarded as a stem cell marker for various healthy as well as cancerous cells. Patient-derived neural and glioma stem cells were historically isolated on the basis of this marker [10,35,36]; however, many controversies surround the use of CD133 solely as a GSC marker, including studies showing that even CD133− cells can eventually give rise to CD133+ cells and form tumors in vivo [37,38]. Furthermore, some fresh GBM specimens do not exhibit detectable levels of CD133 [39]. Interestingly, transcriptional profiles of CD133+ and CD133− GSC lines suggest different cells of origin [40]. These observations indicate that a single marker such as CD133 is not suitable for GSCs isolation. More recently, GSCs are increasingly isolated based on the expression of a variety of surface markers, such as CD24 and/or SSEA-1 (CD15) [41,42]. However, none of these markers have been shown to sufficiently confer stem cell-like properties [43,44]. Until further data are found to support the use of multiple GSC markers, we recommend culturing patient-derived cells directly as neurospheres with no further selection/purification as long as they pass the quality control of “stem-like cells”, including multipotency, ability to self-renew and to differentiate into multiple cell lineages.

### 2.2. Sphere Formation Assays

Presence of cancer stem cells (CSCs) in leukemia has been known for decades [45], but their occurrence in solid tumors was part of a debate until these cells were discovered in breast and brain tumors [46,47]. These landmark findings were possible thanks to identifying and utilizing stem cell markers and their properties. The discoveries made with CSCs highlighted the importance of self-renewal assays and made them a go-to gold standard tool to identify CSCs [48,49]. Many different assays have been developed to evaluate GSC stemness, each focusing on a specific aspect of sphere formation, but most exploit the self-renewal ability of these cells. Anchorage-dependent cells undergo anoikis, a form of programmed cell death caused by loss of cell adhesion under anchorage-free conditions [50]; however, GSCs can grow in a suspended serum-free environment. Utilizing this property, sphere formation assays allow for the assessment of stemness and clonogenic potential of GSCs in vitro.

**Figure 2:**
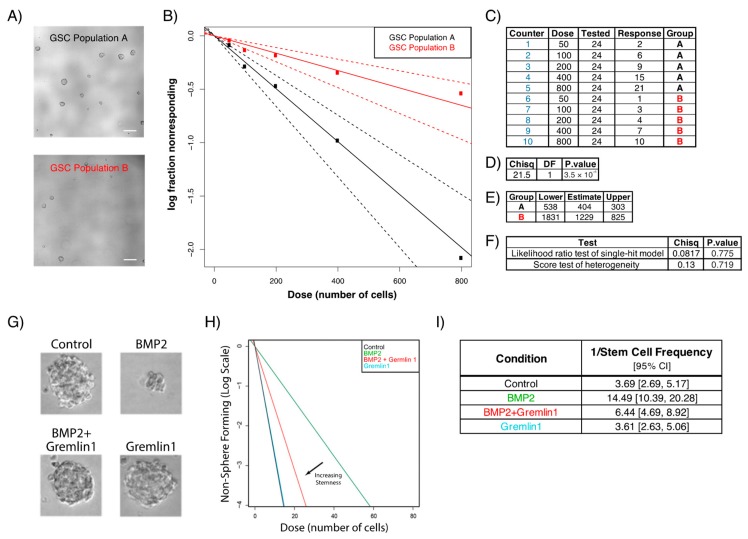
In vitro analysis of GSC self-renewal using the limiting dilution assay. GSCs are dissociated into single cells and different amounts are plated in different wells. The number of wells containing spheres is then evaluated and micrographs are obtained to visualize sphere morphology. In the examples provided, sphere formation assays are performed on different GSC populations to evaluate their stemness and clonogenic potential. (**A**) Micrographs of GSC population A (top) and GSC population B (bottom); scale bar, 200µm. (**B**) The amount of initially seeded cells (*x*-axis) is plotted against the log fraction of non-responders corresponding to wells without any detected spheres (*y*-axis). The slope of the line represents the log-active cell fraction. (**C**) Data obtained using the limiting dilution assay entered into the extreme limited dilution assay (ELDA) tool. (**D**) Overall test for differences in stem cell frequencies between the two groups. (**E**) Confidence intervals for 1/(stem cell frequency). (**F**) Goodness of fit tests. (**G**) Representative images of neurospheres formed by 3565 GSCs following 10 days of Bone Morphogenetic Protein 2 (BMP2) and/or Gremlin1 treatment. (**H**) In vitro limiting dilution assay and (**I**) quantification following 10 days of BMP2 and/or Gremlin1 treatments. (**G**–**I**) Reproduced and adapted with permission from Yan et al., Genes & Development; published by Cold Spring Harbor Laboratory Press, 2014 [51].

One of the most common ways to assess self-renewing frequencies of GSCs is the limiting dilution assay using linear regression analysis. This method has become the gold standard to evaluate GBM stemness and to test novel therapeutics. In brief, GSCs are dissociated and different amounts of cells, down to a single cell, are plated in ultra-low adherent culture plates. GSCs are then treated with the compound of interest and the number of wells containing spheres is quantified around 5 days later. Figure 2 shows phase-contrast images of different groups (Figure 2A,G), as well as sample analysis using the extreme limiting dilution program (http://bioinf.wehi.edu.au/software/elda/), although any statistical software can be used (Figure 2B,H). In the example provided in Figure 2H, the authors used this method to evaluate the effect of Bone Morphogenetic Protein 2 (BMP2) alone or in combination with Gremlin1, where the *x*-axis represents the amount of initially seeded cells and the *y*-axis shows the log fraction of non-responders (wells where no sphere was detected) [51]. This tool provides valid goodness of fit tests to examine stemness of any given cell population (Figure 2C–F,I) [52].

**Figure 3:**
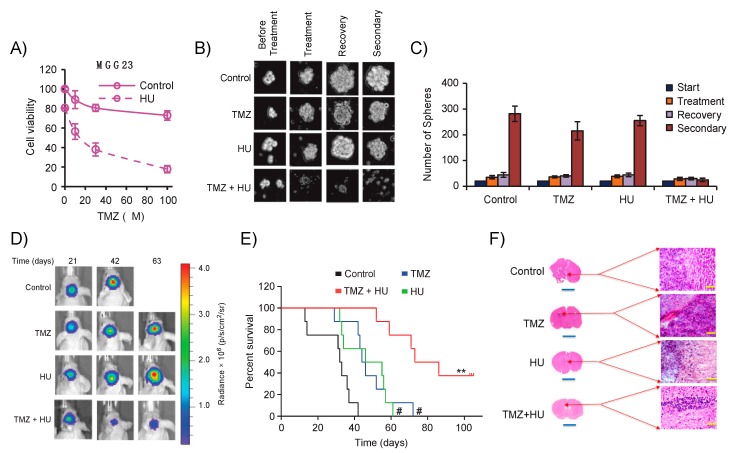
Tumor xenograft analysis workflow in vitro, in vivo, and ex vivo. (**A**) Patient-derived MGG23 GSCs were treated with different concentrations of temozolomide (TMZ; 100 nM to 1 mM) in the presence or absence of 30 μM hydroxyurea (HU), and cell viability was measured 3 days post-treatment. Results are shown as the mean ± SD where the vehicle control was set at 100%. (**B**,**C**) MGG23 neurospheres were treated with either DMSO, HU, TMZ, or HU + TMZ for 4 days. Spheres were counted, washed, and left without treatment for another 5 days to allow recovery. Recovered spheres were dissociated and plated in a new 48-well plate to measure secondary sphere formation 5 days later. Representative image from each treatment group is shown; scale bar, 200 μm (**B**). Total sphere numbers in the well were recorded at each event (**C**). (**D**,**E**) Mice-bearing MGG23 tumors were injected with DMSO, HU, TMZ, or TMZ + HU. Representative Fluc bioluminescence image of a single mouse from each group is shown over time (**D**) with corresponding Kaplan–Meier survival analysis comparing the two groups; # *p* < 0.05 vs. control, ** *p* < 0.01 TMZ + HU vs. TMZ (**E**). (**F**) Ex vivo histological analysis with Haematoxylin and Eosin staining 42 days after tumor injection. Reproduced and adapted with permission from Teng et al., Neuro-Oncology; published by Oxford University Press, 2018 [53].

An alternative approach to the limiting dilution assay to analyze GSCs self-renewal is to monitor sphere formation, recovery, and secondary sphere development. Here, GSCs are treated with a drug of interest for 3–5 days, and cell viability as well as sphere size is analyzed. The drug is then washed off and sphere recovery is monitored over time. Recovered spheres are then dissociated, re-plated in new wells, and secondary sphere formation is monitored again over time. Recently, we have shown that the US Food and Drug Administration (FDA) approved compound hydroxyurea sensitizes GSCs to temozolomide [53]. In the example provided in Figure 3, GSCs are treated with either temozolomide and/or hydroxyurea, and the sensitization effect of the latter on GSCs is monitored by measuring the number and size of spheres in each well at each time point (Figure 3A–C) [53].

## 3. In Vivo GBM Model

Patient-derived xenografts (PDX) or patient-derived cancer (stem) cells are widely used models in cancer research, particularly in neuro-oncology. To create an in vivo tumor model, cells are either directly inoculated from patients into immunocompromised mice, or first cultured in vitro, where they can be subjected to genetic modifications prior to implantation. PDX models provide the possibility of studying cancer growth, treatment response, and survival outcome in a living animal.

### 3.1. Establishing Patient-Derived Xenograft GBM Model

All animal studies should first be approved by the Institutional Subcommittee on Research Animal Care following guidelines set forth by the National Institutes of Health Guide for the Care and Use of Laboratory Animals. In brief, immunocompromised athymic nu/nu mice (male or female) are anesthetized with isoflurane or a mixture of ketamine (100 mg/kg ketamine and 5 mg/kg xylazine) prior to implantation. Once mice are under anesthesia, a small cutaneous cut is made on their heads, and lidocaine with epinephrine is applied locally to control pain and bleeding. For GBM models, the following coordinates are used for implantation into the striatum with respect to the bregma: X (lateral) = 2.0, Y (frontal) = 1.0, Z (ventral) = −2.5. GSCs are usually stereotactically implanted in different amounts (depending on the model) as small spheres (typically dissociated the day before surgery) in 2 µL phosphate-buffered saline (PBS) using a 30-gauge Hamilton syringe. Using a microsyringe pump controller, 2 μL of cell suspension is injected at a rate of 1 μL/min. After the injection is complete, the needle is withdrawn about 0.3 mm every 5 min to ensure optimal implantation and avoid backflow of the injected cells through the needle tract [54].

### 3.2. PDX Mirrors Hallmarks of Parental Tumor

GBM is known for its inter- and intratumoral heterogeneity, including diverse histological patterns and cytological hallmarks. These characteristic features of GBM are of clinical relevance when evaluating predictive therapy. As we begin to better understand GBM, the phenotype and genotype of a particular tumor must be taken into account in order to provide optimal and targeted personalized therapy. GSCs have been recognized as tumor-initiating cells, and the driving force for invasion/migration, recurrence, and therapeutic resistance [11]. Murine models using patient derived GSCs have been shown to mimic many aspects of the parental tumor. Wakimoto et al. (2009) described how human-derived GSCs are able to efficiently generate tumors that invade the brain upon intracranial implantation into immune-deprived mice [34]. Not only does this model mirror the invasiveness of GBM, but it further exhibits other common features of patient tumors. For example, some patient-derived GSCs, such as GBM8 and GBM6, spread from one brain hemisphere to the opposite hemisphere via the corpus callosum. The GBM8-based model also showed a butterfly-like growth pattern, a pre-eminent characteristic of GBM, and tended to expand alongside the subventricular areas, leading to a compression of the lateral ventricles. All GSC lines were able to recapitulate histological hallmarks of the original tumors, including pseudopalisading necrosis, invasiveness, and increased angiogenesis [11,34].

In addition to PDX mirroring primary tumor pathology, these models recapitulate subtype-specific growth patterns. For instance, GSCs obtained from the aggressive MES subtype grow at a much higher rate upon implantation compared with the PN subtype, manifesting in higher invasiveness and increased vascularity [25,55]. Thus, the genetic background and/or original molecular subtype of GSCs should be taken into consideration when deciding on the number of cells needed to form tumors in xenograft models. For instance, as few as 50 cells are able to generate lethal tumors using GBM8 GSCs harboring copy number amplification of platelet-derived growth factor receptor alpha (PDGFRA) and MYCN as well as cyclin-dependent kinase Inhibitor 2AB (CDKN2AB) homozygous deletion [34].

### 3.3. Bioluminescence Imaging from In Vitro to In Vivo

Imaging has been one of the fastest rising fields in science, and many studies are underway to improve and develop new means of imaging tumor development, progression, invasion/metastasis, plasticity, as well as transcriptional activation and processes within the tumor, non-invasively. Apart from imaging techniques commonly used in the clinic (X-ray, computed tomography, magnetic resonance imaging, or ultrasound), drastic improvements in molecular imaging have been accomplished both for pre-clinical and clinical analysis. With the discovery of different luciferases and the design of new reporter proteins, we are able to visualize and follow up biological processes non-invasively, both in vitro and in vivo. Although many luciferases [*Firefly* luciferase (Fluc), *Renilla* Luciferase (Rluc), *Vargula* luciferase (Vluc), and *Gaussia* Luciferase (Gluc)] have been described, Fluc remains as the most commonly used reporter for in vivo monitoring of tumor growth and response to therapy because of the convenient intraperitoneal administration of its substrate. On the other hand, the Gluc reporter is naturally secreted, thus tumor/GSCs growth, proliferation, and response to therapy can be assessed by monitoring its activity in an aliquot of conditioned medium in culture or in blood ex vivo, keeping cells and animals intact for validation analyses [56]. Since Fluc, Gluc, and Vluc utilize different substrates, they can be multiplexed together in the same system to monitor three distinct biological processes within the tumor or its microenvironment simultaneously, such as different subpopulations of tumor cells and their response to therapy, or activation of different pathways, both in culture and in vivo [57,58]. For instance, to monitor cell viability, the reporter of choice is cloned under the control of a constitutively active promoter such as the cytomegalovirus or SV40 promoter. To monitor activation of transcription factors/pathways such as Nuclear factor kappa-light-chain-enhancer of activated B cells (NFkB), the reporter of choice is cloned under the control of NFkB responsive elements [57]. The most effective way to engineer GSCs to express these reporters is to use lentivirus vectors, as adding the vector directly to the conditioned medium of GSCs results in >90% transduction efficiency and allows for integration of the expression cassette into the genome of cells, yielding stable expression of the reporter [59,60,61].

#### Evaluating Novel Therapeutics against GSCs from Dish to Animals

Once GSCs are injected into the striatum, tumors are formed reproducibly, invading the brain of mice in a similar manner to human GBMs, within several weeks, depending on a multitude of factors, including the number of cells implanted as well as the molecular subtype of the tumor. For instance, PN GSCs exhibit much slower tumor growth compared with the more aggressive MES subtype [17,25]. Tumor growth is typically imaged once a week with bioluminescence imaging in cases where GSCs are pre-engineered to express a luciferase reporter. For example, if the tumor expresses Fluc, mice are intraperitoneally injected with the d-Luciferin substrate and imaged 10 min post-injection using a cooled charge-coupled device camera (such as the IVIS imaging system). The tumor-associated signal is linear with respect to tumor volume and therefore can be used as an easy means to monitor GBM growth and response to therapies non-invasively, complementing survival analyses [53,60,62]. In the example provided in Figure 3, we used this method to assess the therapeutic efficacy of hydroxyurea as adjuvant therapy for GBM in combination with temozolomide and showed that the addition of hydroxyurea inhibited tumor growth and extended overall survival (Figure 3D–H). An important point when assessing multipotency is to implant a very low number of GSCs, such as the limiting dilution analysis described in Figure 2, and observe potential tumor growth [34,63]. When using the more aggressive MES GBM subtype, PDX will grow and form a tumor with as low as 50 injected cells [34]. A possible hurdle, however, could be the detection of the initial tumor by bioluminescence imaging due to the sensitivity of this technique. One would have to rely on survival and ex vivo histological analyses to appreciate tumor formation and propagation. Alternatively, the secreted Gluc reporter and ex vivo blood analysis could be used in place of imaging, since this assay can detect as few as 1000 implanted cells [60,64].

### 3.4. Murine Cancer Models as Pre-Clinical Platforms for Cancer Studies and Drug Therapy

A large number of rodent models have been developed to study initiation and progression of cancer and have served as preclinical platforms to evaluate drug response. The most commonly used models are transgenic or genetically engineered mice (GEM) and patient-derived xenografts. Each model comes with its benefits and shortcomings (summarized in Table 2). For example, GEM models are useful for learning the role of different mutations/oncogenes in stemness, tumorigenesis, and invasion/metastasis. A tremendous amount of information has been gained in neuro-oncology by altering the genetic code of mice either through deletion, mutation, or overexpression of certain genes. Recently ‘*The Cancer Genome Atlas’* (TCGA) data have further increased our understanding of the molecular pathogenesis of GBM. Through genetic analyses, GBM can now be classified into three molecular subtypes (classical, mesenchymal, proneural) on the basis of aberrations or expression levels of certain genes, including epidermal growth factor receptor (EGFR), PDGFR, NF1, and the receptor tyrosine-protein kinase ERBB2 [6,16]. With this in mind, GEM models have been shown to be essential in understanding the pathology of GBM. In one example, authors developed a transgenic mouse model expressing the oncogene v-Ha-ras to evaluate the role of the p21-ras pathway in GBM. [65]. They showed that the level of v-Ha-ras is directly proportional to the development of astrocytomas. Chimeras expressing high levels of v-Ha-ras developed multifocal malignant astrocytes and died within a couple of weeks. Moderate gene level expression led to germ line transition. Cells originated from these astrocytomas were able to induce tumor growth upon inoculation into different hosts. These genetically induced astrocytomas showed numerous similarities to their human counterpart, including anisonucleosis, high mitotic index, infiltration, necrosis, and angiogenesis [65]. Even though these GEM can recapitulate a human tumor in various ways, they are unable to completely reproduce the genetic intricacy present in the tumor. For instance, many types of tumors, including GBM, have an extensive degree of heterogeneity, meaning that the genetic makeup of a cell can be totally different compared to its neighboring cells. It is currently not possible to replicate this phenomenon in GEM models, which may present a large hurdle when attempting to translate therapeutic results from animal to human. When investigating a patient’s response to a specific drug regimen, it is imperative to examine this response in a human tumor and not a mouse tumor. As a result, the use of mouse models incorporating patient-derived xenografts have gained popularity because of their close resemblance to the parental tumor [66]. Notably, PDX have increasingly been used as an integrative platform for pre/co-clinical treatment trials. They allow holistic analyses of genomic profiles and clinical data of a specific tumor tissue, as well as drug screening and drug response testing to determine a patient-specific drug therapy [67]. GBM PDX have further been shown to overcome limitations of conventional established cell lines by predicting clinical success of novel therapeutics. For instance, Bevacizumab was found to have anticancer properties and managed to prolong survival in a xenograft model established with U87 cell line [68]; however, this drug failed to significantly prolong overall survival in a phase 2 clinical trial against GBM [69]. These clinical findings are in line with studies by Joo et al. (2013), showing that bevacizumab did not alter the overall survival using GBM PDX models [70].

Another example is the pre-clinical evaluation of the drug Selinexor, a selective inhibitor of nuclear transport, against GBM [71]. In this pre-clinical platform, multiple patient-derived GBM cells were introduced into nude mice, and treatment with Selinexor was initiated upon tumor formation. Selinexor showed a dose-response growth inhibition in all seven used patient-derived cells in culture and in orthotopic PDX, leading to an increase in overall survival. On the basis of these results, Selinexor went in early clinical trials in patients with relapsed GBM in 2014, and the trial is expected to terminate in December 2019 (www.clinicaltrials.gov.com). Successful examples for the use of PDX does not only apply to GBM, but is extended to other tumor types, such as the use of Herceptin for adjuvant therapy with paclitaxel and doxorubin against breast cancer overexpressing the human epidermal growth factor receptor 2 (Her2/Neu) protein [72,73], and in non-solid tumors such as multiple myeloma, using the proteasome inhibitor Bortezomib/Velcade [74,75,76]. These are only a few successful stories wherein orthotopic PDX in mice sufficed as a valid pre-clinical model.

### 3.5. Phenocopying GBM Intratumoral Heterogeneity from Patients to Xenografts

One of the key features of GBM is intratumoral heterogeneity. Different cell populations can be found within the same tumor, whereby each cell might express a different degree of differentiation and exhibit unique genetic mutations. The question remains whether, once removed from the patient tumor, GSCs maintain their original molecular and cellular profile and continue to resemble the parental tumor. A study conducted by Wakimoto et al. (2011) looked into preservation of the phenotype and genotype of the primary tumor upon xenotransplantation. The group established a panel of GSCs derived from 15 newly diagnosed GBMs. To analyze the degree of resemblance between the patient’s tumor and corresponding xenografts, they conducted phenotypic and genomic characterizations of both original GSCs and the orthotopic tumors [77]. Overall, similarities were evident between the patient–xenograft pairs, including histological features of GBM such as giant cells, fibrillary processes, and oligo-like cells; however, few mismatches were found when examining endothelial proliferation and necrosis. Most xenografts did not portray necrosis, a typical feature of GBM, which might be due to the relatively smaller size of the mouse tumor compared to the human tumor. Genomic profiling identified several copy number alterations in different GBM subtypes as well as discrepancy between the GSCs and their corresponding patient tumor. One possible explanation is that GBMs may contain a selection of different GSC subsets within the tumor, and a particular subset might be able to adapt and proliferate better in cell culture conditions. Another possible explanation is that the isolated GSCs used for the analysis and the fresh frozen sample were derived from a different tumor site. This could lead to discordant results, as the tumor is very heterogeneous and the findings only represent a small area of the whole tumor.

### 3.6. A New Era for GBM Models?

Currently, GBM research rests mainly on human tumor cell lines, patient-derived tumor initiating cells, and orthotopic PDX models. There are major concerns regarding the use of tumor cells lacking the tumor (micro) environment, and of PDX models being mostly established in immunodeficient mice to allow graft survival. In the last couple of years, the use of human slice cultures, also known as organotypic slice cultures, emerged as a better approach to study disease and therapy. In 2013, a research group in Germany demonstrated for the first time the specific effect of radiation and TMZ on human GBM tissue ex vivo [78]. Tumor tissues were directly obtained from different patients undergoing surgical tumor resection and were immediately transported to the laboratory where they were sectioned and maintained in cell culture plates (on a specific membrane) for up to a month. These tissue slices were subjected to different therapy regimens including high intensity radiation and TMZ. The authors showed that high intensity carbon ions and photons significantly decreased proliferation and induced DNA damage in a time-specific manner. Another interesting finding was that TMZ might be effective in tumors despite their DNA repair enzyme *O*-6-methylguanine-DNA methyltransferase (MGMT) status. These data demonstrate that tumor-derived GBM slice cultures could be used as a novel approach for testing new therapeutics in a clinically relevant environment.

An additional aspect that contributes to the aggressiveness and poor survival of GBM is the diffuse infiltration of GSCs into the neighboring healthy tissue [79,80]. The majority of studies evaluating tumor cell migration use scratch, gap closure, and/or Boyden chamber assays. Although these methods are easy, reproducible, and inexpensive, recent studies have demonstrated the importance of 3D models when analyzing tumor cell migration/invasion, as they better represent the natural GBM microenvironment [81,82]. In their natural habitat, tumor cells are embedded in a matrix consisting of the extracellular matrix and multiple cell types, each playing their own role in supporting the tumor microenvironment. It has become clear that an invasion assay should closely imitate the natural milieu of glioma tumor cells [83]. The current state-of-the-art to assess glioma invasion is utilizing organotypic slices, which can be cultured for several days to weeks without losing their cellular structure. Previous studies have used the organotypic slice culture as a novel tool to study migration and invasion of ex vivo implanted tumor cells [84,85]. Eisenmann et al. (2018) demonstrated that by using adult mouse brains instead of perinatal mouse brains (the most frequently used) as donors, they could implant any type of cells and follow migration with more precision and accuracy. This is due to the fact that the adult mouse brain is able to better recapitulate the physiological state in vivo (adult mouse brains have full myelination and a firm extracellular matrix, in contrast to young mice). They also demonstrated that their protocol allowed manipulation of various factors, including the use of exogenous small molecule treatment and genetically manipulated mice as donors for the organotypic slices, allowing better analysis of the role of specific genes in tumor cell migration and invasion [86]. In summary, the described practical applications illustrate the efficacy and flexibility of organotypic slice cells as new advanced ex vivo GBM models as well as their applicability in testing novel therapeutics in a clinically relevant environment.

A more recent approach to model GBM in vitro utilizes cerebral organoids, or so-called ‘mini-brains’, generated using pluripotent stem cells, that, after addition of an intricate sequence of media and additives, slowly grow and differentiate. To allow for optimal growth, cerebral organoids are eventually cultured in rotating bioreactors where they grow to contain functionally mature populations of all brain cell types and further mirror the regional differences of the human brain [87]. Ogawa et al. (2018) demonstrated the use of cerebral organoids to model and study GBM development and pathology [88]. They showed that tumorigenesis could be initiated by both genetically engineering cerebral organoids (altering the fourth exon of TP53 and introducing an oncogenic HRas virus) and by injecting organoids with patient-derived GSCs. Bian et al. (2018) used a similar approach and introduced different oncogenic mutations in cerebral organoids to recapitulate brain tumorigenesis and to study GBM pathology and evaluate drug response [89].

Another exciting new “fresh off the press” approach is glioblastoma on a chip. Hee-Gyeong et al. (2019) embedded patient-derived GBM cells together with endothelial cells in a decellularized extracellular matrix from a porcine brain, resulting in an ex vivo model that displays most properties of the parental tumor [90]. They successfully demonstrated that the GBM-on-a-chip mirrors patient treatment resistance, suggesting this model as a promising future personalized therapy-screening platform. However, GBM-on-a-chip model is missing one of the most important factors in GBM, the immune cells. Authors speculate that eventually they could build an immune component into the same system.

In place of murine models, zebrafish started to gain popularity. Zebrafish was first introduced in 2005 by Lee et al. as a cancer model when the group successfully implanted human melanoma cells in zebrafish embryos [91]. Subsequently, many zebrafish-based models with a wide variety of human cancers have emerged [92,93], including GBM [94,95,96]. These models have many advantages: (1) zebrafish physiologically resemble humans, (2) they are easy to handle and inexpensive in comparison to murine models, (3) they are good predictive models for high-throughput drug screening, (4) they are transparent in their early stage, allowing easy visualization of tumor progression and treatment response, and (5) they multiply fast and are easy to maintain, resulting in a higher number of embryos that can be used to achieve a greater power and significance [97]. Xenografts are usually established during the embryonic stage, which allow the tumor to engraft in an efficient manner, as the adaptive immune system has not yet been fully developed. On average, it can take up to 21 weeks for tumors to form in murine models; however, in zebrafish, tumor engraftment can take place in just a few days [97,98]. Moreover, anticancer drugs can be easily tested in this model, as they are able to absorb small molecules straight from their environment [99]. To avoid cues from the environment that might change the tumor phenotype, some reports have suggested implanting the tumor cells into the yolk sac [91], where cells are able to proliferate in an acellular nutrient rich environment without space limitations [100]. There are different ways to track tumor growth in zebrafish. The most popular method is enzymatic dissociation of the larvae, followed by manual counting of the labeled cells with a hemocytometer [100], or by semi-automated counting on the basis of micrographs [101]. The proliferation marker Ki-67 has been recently used for the same purpose by Pudelko et al. (2018) to show tumor cell division in GBM [102]. Further, flow cytometry can be applied to monitor cell proliferation [103]. Finally, a method that has been gaining ground is the light sheet fluorescence microscopy. Compared with standard confocal systems, this method has low phototoxicity, allowing imaging of zebrafish for a longer period of time, and has been used successfully to follow GBM growth and therapeutic response [102]. Although simultaneous use of these modalities has been successful for GBMs [104], several factors need to be considered; zebrafish embryos usually grow at 28 °C, a much lower temperature than mammalian cells. Various studies using GBM xenografts reported working with temperatures between 25 and 36 °C, with a survival rate between 87.5 and 95% [105]. Although tumor cells are usually implanted in the yolk sac, a study published by Zeng et al. (2017) showed that GBM cells could also be implanted in the zebrafish brain, mimicking the GBM environment in a clinically relevant scenario [106].

## 4. Discussion

With the growing need of biologically relevant cancer models allowing scientists to study the disease outside the host and to find novel therapeutics using different techniques, PDX began to grow in popularity around two decades ago. Since CSCs come directly from human tumor specimens, these models are thought to recapitulate the actual disease more closely, allowing for the study of tumor behavior and etiology in a more accurate manner. However, like any other cancer model, PDX has its advantages as well as its shortcomings compared to established cell lines. On one side of the spectrum, established cancer cell lines are cheaper, easier to handle, and the experiments conducted on them are more reproducible. However, many studies have shown that tumors from established cancer cell lines have adapted to their artificial environment and thus the acquired mutations do not resemble the original tumor [107]. Furthermore, it is often unclear which cancer subtype these cells originate from, and to what extent they resemble the tissue of origin. A landmark study published by Domcke et al. in 2013 attempted to answer these questions [108]. The group reviewed genetic resemblances and discrepancies between ovarian cancer cell lines and “real” tumors. By examining the genetic information of ovarian cancer cell lines in the *Cancer Cell Line Encyclopedia* (CCLE) and the genomic data of ovarian tumors in TCGA, they concluded that the most commonly used ovarian cancer cell lines resembled the real tumors very poorly, while cell lines displaying the biggest overlap with the original tumor were rarely used [108]. Moreover, Allen et al. (2016) compared their original U87MG GBM cell line with the same cell line distributed from the American Type Culture Collection (ATCC) and Cell Line Service (CLS) [109]. By running a short tandem repeat analysis (STR) and examining the mitochondrial DNA, they discovered that the original U87MG did not genetically match the U87MG provided by ATCC and CLS, even though the first U87MG cell line came from the same institution. Although analyses have demonstrated that these cells are of CNS origin, they did not fully match the reference line of origin, suggesting a mix-up or contamination over the years [109]. In addition, Lee et al. (2006) showed that the transcriptome of conventional glioma cell lines grown in serum, including U87MG and U251MG, do not correspond to those of glioma tumors and are poor tumor representatives [21].

GBM is defined by a complex and versatile genetic landscape with inter- and intratumoral heterogeneity [110]. Wakimoto et al. (2012) demonstrated that GSCs can generate cell lineage differentiation patterns mirroring the original patient GBM and can form tumors consisting of a mix of cytologically heterogenous cells [77]. Furthermore, patient-derived GSCs have been utilized to help generate an integrative model of cellular states and used to show genetics and plasticity of GBM using single-cell RNA-sequencing [8,111].

Through a project funded by the National Institutes of Health, the Mayo clinic now has a repository of different patient-derived cells available through a simple material transfer agreement to all academic researchers (https://www.mayo.edu/research/labs/translational-neuro-oncology) [112,113,114]. This repository allows scientists to acquire cells on the basis of their GBM subtype (defined by RNAseq or methylomics) and to filter their selection on the basis of a plethora of parameters, including molecular subtype, EGFR amplification/mutation, MGMT methylation, or isocitrate dehydrogenase 1 and 2 (IDH1/2) gene status. This could be of importance, since the somatic mutations found in the IDH1/IDH2 gene are mostly found in low grade gliomas (LGG) and the proneural GBM subtype [6,115]. IDH genes are involved in oxidative and reductive metabolism of the cell. Mutation of this gene leads to the generation of the oncometabolite (*R*)-2-hydroxyglutarate, which causes epigenetic modifications and tumor formation [116]. Recently, it has been reported that intermediate vascular abnormalities are seen in lower grade gliomas. More interestingly, IDH-wild-type LGG harbors a specific vascular expression pattern when compared to IDH-mutated LGG. This could present as a new therapy target for IDH-wild-type patients [117]. Early identification of IDH mutation and MGMT status is clinically relevant, as it impacts prognosis and treatment [118]. Patients harboring IDH1 mutations and displaying a methylated MGMT status showed improved overall and progression-free survival compared to patients with IDH wild type and unmethylated MGMT [119]. IDH mutations have been linked to the methylation status of the MGMT gene and play an important role when assessing TMZ response. Integration of these findings in PDX models by selecting the appropriate patient-derived cell line is essential in developing models to better understand GBM pathology.

PDX models are not flawless, and researchers are beginning to identify their shortcomings. It has been clear for some time now that genetic changes do occur because of long-term culture in changed environment and conditions, and the question remains as to if and how these mutations could have an effect on experimental outcomes. Another major issue is that these PDX models, which require an immunocompromised environment, are not typically suited to study tumor immunology and immunotherapy, unless they are used in the context of a humanized mouse model, which could be challenging on its own [120]. Keeping in mind that the perfect model does not exist, the most straightforward, reliable, and reproducible platform currently available to study GBM are these GSCs, as they are derived from patients and resemble the tumor-of-origin the most. However, one should always consider the shortcomings discussed above when using these models.

## Figures and Tables

**Table 1:** Cancer stem cell cultures compared to established cell line cultures.

	Cancer Stem Cell	Established Cell Line
**Advantages**	Retains tumor heterogeneity	Highly time and cost effective
	Mimics phenotype of original tumor	Easy to obtain and culture
	Recapitulates genetic, epigenetic, proteasomal, and transcriptomal make-up of original tumor	
	More likely to form adequate tumors in vivo	
**Disadvantages**	Cost- and labor-intensive	Artificial selection in vitro
	Only low number of passages recommended	Lack of tumor architecture and heterogeneity
	Potential low take and growth rate of patient-derived xenografts (PDX)	Does not mirror clinical response
		Does not show single-cell invasion, tumor necrosis, or microvascular proliferation
		Does not mirror genotype of original tumor

**Table 2:** Genetically engineered mouse (GEM) models compared to orthotopic PDX models.

	GEM Models	Orthotopic PDX Models
**Advantages**	Mice are immunocompetent	Better predictors of therapy efficacy in patients
	Customized genetic modifications	Biomarkers for targeted therapy can be identified
	Tumor development can be followed over time	Heterogeneity is preserved
**Disadvantages**	Mutations are limited, cannot reproduce heterogeneity	Cannot customize mutations
	Mouse tumors are not a good predictable model for human therapy response	Mice are immunocompromised. Not suited to study immunotherapy
